# Insights into Lipid-Based Delivery Nanosystems of Protein-Tyrosine Kinase Inhibitors for Cancer Therapy

**DOI:** 10.3390/pharmaceutics14122706

**Published:** 2022-12-03

**Authors:** Josef Jampilek, Katarina Kralova

**Affiliations:** 1 Department of Analytical Chemistry, Faculty of Natural Sciences, Comenius University, Ilkovicova 6, 842 15 Bratislava, Slovakia; 2 Institute of Neuroimmunology, Slovak Academy of Sciences, Dubravska Cesta 9, 845 10 Bratislava, Slovakia; 3 Institute of Chemistry, Faculty of Natural Sciences, Comenius University, Ilkovicova 6, 842 15 Bratislava, Slovakia

**Keywords:** protein-tyrosine kinase, tyrosine kinase inhibitors, anticancer, nanoparticles, self-nanoemulsifying drug delivery systems, nanoemulsions, liposomes, solid lipid nanoparticles, lipid-polymer hybrid nanoparticles, nanostructured lipid carriers

## Abstract

According to the WHO, cancer caused almost 10 million deaths worldwide in 2020, i.e., almost one in six deaths. Among the most common are breast, lung, colon and rectal and prostate cancers. Although the diagnosis is more perfect and spectrum of available drugs is large, there is a clear trend of an increase in cancer that ends fatally. A major advance in treatment was the introduction of gentler antineoplastics for targeted therapy–tyrosine kinase inhibitors (TKIs). Although they have undoubtedly revolutionized oncology and hematology, they have significant side effects and limited efficacy. In addition to the design of new TKIs with improved pharmacokinetic and safety profiles, and being more resistant to the development of drug resistance, high expectations are placed on the reformulation of TKIs into various drug delivery lipid-based nanosystems. This review provides an insight into the history of chemotherapy, a brief overview of the development of TKIs for the treatment of cancer and their mechanism of action and summarizes the results of the applications of self-nanoemulsifying drug delivery systems, nanoemulsions, liposomes, solid lipid nanoparticles, lipid-polymer hybrid nanoparticles and nanostructured lipid carriers used as drug delivery systems of TKIs obtained in vitro and in vivo.

## 1. Introduction

Tumors of various etiology are one of the most common reasons for disease occurrence and deaths. According to the WHO, breast, lung, large bowel, rectal, and prostate cancer are the most frequent. All over the world, cancer caused almost 10 million deaths in 2020, i.e., every sixth death [1]. High-income regions have twice the number of malignancies than low-income countries, mainly due to unhealthy lifestyle (tobacco overuse, high body mass index, alcohol consumption, low vegetable and fruit intake, insufficient physical activity, and stress), while approximately 30% of cancer cases in low- and middle-income countries is caused by infections (e.g., by human papillomavirus and hepatitis). Even hereditary predisposition cannot be excluded (e.g., in breast cancer) [1,2].

It is very hard to determine exactly the time for antitumor treatment start, because plant preparations have been continuously used since ancient times [3]. The beginnings of modern anticancer chemotherapy (cancer cell proliferation inhibition) can be found in the 1940s [4]. This idea was based on the observation of sailors exposed to yperite after the Luftflotte 2 air raid on Bari during World War II. Gilman et al. noticed considerable hypoplasia of the lymphoid and myeloid cells of one of the sailors exposed to the effects of mustard gas. Subsequently, different derivatives, or yperite analogues, so-called *N*-yperites, were developed [5,6]; some of them have been used as cytostatics until now. For example, in 1959, the U.S. FDA approved cyclophosphamide [7]. Another crucial milestone in cytostatic development was the discovery of the essential role of folic acid [8] in DNA metabolism [9,10]. Sidney Farber hypothesized that folate antagonists should be able to inhibit tumor growth and slow down disease progression [11] and published a study in 1948 elucidating the effects of folate antagonists [12]. Besides, Farber first described the compound known as methotrexate [13]. Thus, the first possibility of the treatment of acute leukemia—the disease that was believed to be incurable—arose. Unfortunately, Farber did not find collective understanding in his time; however, already in 1958, employees of the U.S. National Cancer Institute (NCI), Hertz & Li, reported the curing of solid tumor choriocarcinoma by methotrexate [14]. Farber’s work and the antifolate idea gave rise to the thesis of G. Hitchings and G. Elion from Burroughs Wellcome Co., (today GlaxoSmithKline) [15], who dealt with a similar thought as Farber, that small changes in physiological molecules can lead to large changes in the physiological properties of these compounds and thus inhibit cancer cell growth by their ability to interfere in de novo RNA and DNA synthesis [16]. This way, antimetabolites on the basis of purines (6-mercaptopurine) came into existence [17]. In a paper from 1954, Elion & Hitchings described the effects of 6-mercaptopurine and a combination of 6-mercaptopurine with methotrexate on adenocarcinoma, sarcoma, and leukemia cells [18]. Classic antimetabolites of thymine, a pyrimidine base, include 5-fluorouracil that was patented by C. Heidelberger in 1957 and ranks among the most used cytostatics at present [19]. It should be noted that in 1955, the National Chemotherapy Program was first initiated in the NCI in the U.S., and thus, the systematic screening of new drugs began [14,20].

In the course of previous decades, several main groups of cytostatics were developed, including different types of alkylating agents (*N*-yperite analogues, alkylsulfonates, ethylenamines, nitrosoureas, epoxides, and other alkylating agents), folic acid derivatives, alkaloids and plant medicines (Vinca alkaloids, podophyllotoxin or colchicin derivatives, taxanes, and other natural products), cytotoxic antibiotics (actinomycins, anthracyclines and related substances, and other toxic antibiotics), complex-forming compounds from the group of platinum cytostatics, and methylhydrazines [21,22]. One more group is of the various sensitizers used in photodynamic or radiation therapy. A big anatomical therapeutic chemical (ATC) [22] classification group is the so-called other antineoplastic agents, e.g., hydroxyurea, estramustine, topotecan, etc. [21]. The discovery of new antiviral agents is also associated with the research and development of new cytostatics (from the groups of purine and pyrimidine analogues) [21,22]. The development of biology, physiology, and chemical sciences enabled the origination and synthesis of monoclonal antibodies, e.g., rituximab, trastuzumab, cetuximab, bevacizumab, and others [23,24]. One of the youngest groups is protein kinase inhibitors, which became one of the most important groups of antineoplastic agents soon after their discovery [21,25]. Targeted treatment with monoclonal antibodies and protein kinase inhibitors, as well as their preparation, development, and production were enabled primarily by the development of molecular biology and the ability to decode and model enzyme amino acid sequences.

A significant number of protein kinase inhibitors was developed, and it can be said that they caused a revolution in oncology and hematology over the last 20 years; however, save for several cases of chronic myeloid leukemia, no one patient was cured with only monotherapy [26,27,28]. Problems of the emergence of resistance to treatment and toxicity, leading to a reduction of the administered dose or even treatment discontinuation are main challenges of their use in oncology patients [27,28,29,30,31]. Besides the design of new molecules [27,28,29,32], scientists have developed sophisticated reformulations [33,34] and nanosystems [35], enabling controlled and targeted drug delivery and expecting a quick overcoming of pharmacological and pharmacokinetic difficulties as compared to the development of new original protein kinase inhibitors [36,37,38,39].

This review, in addition to an insight into the history of chemotherapy and a brief overview of the development of tyrosine kinase inhibitors (TKIs) for the treatment of cancer and their mechanism of action, summarizes the results of applications of self-nanoemulsifying drug delivery systems (SNEDDSs), nanoemulsions (NEs), liposomes, solid lipid nanoparticles (SLNPs), lipid-polymer hybrid nanoparticles (LPH NPs), and nanostructured lipid carriers (NLCs) used as drug delivery systems of TKIs.

## 2. Protein Kinase Inhibitors

At the end of the 1980s, molecular and genetic approaches allowed better understanding of cell biology and thus, the discovery of signaling networks regulating such activities as proliferation and cell survival. It was discovered that such signaling networks are considerably changed in cancer cells. This turning point in cell biology ushered in the rise of a new approach in anticancer chemotherapy—targeted therapy [40]. Thus, growth factors, signaling molecules, cell-cycle proteins, apoptosis modulators, and angiogenesis-related molecules became targets of new drug substances [14,31,41]. An example of such a substance is imatinib (IMA) [42], developed in the second half of the 1990s [43,44] and approved for the treatment of chronic myeloid leukemia as the first representative of protein kinase inhibitors in 2000 [45]. In 1961, its discovery was preceded by the identification of chromosomal translocation t(9;22), known as Philadelphia chromosome [46], the result of which was the construction of a fusion gene of breakpoint cluster region–Abelson tyrosine kinase (BCR-Abl tyrosine kinase) [47]. IMA is the first and basic molecule in the protein kinase inhibitor group [42]. It is not a selective inhibitor of BCR-Abl tyrosine kinase, but it inhibits other so-called non-receptor tyrosine kinases [48,49] (see below).

### 2.1. Proteins with Kinase Activity

Kinases belong to the transferase group catalyze phosphorylation, which is a common covalent modification [50] regulating the functionality of proteins. Binding of a phosphate, i.e., a group with strong negative charge, to protein considerably influences its conformation and functions. Phosphorylation (and the opposite process, dephosphorylation catalyzed by phosphatases) serves as an activity switch of a particular protein. Many signaling pathways use kinases and phosphatases as their regulators. These signaling pathways are necessary for signal transduction and cell activity regulation [51]. Thus, kinases control many cell processes, including transcription, cell-cycle process, cytoskeleton reorganization, movement, differentiation, and, especially, apoptosis [52,53,54,55]. Therefore, mutations and dysregulation of these protein kinases play a casual role in some human diseases [26,54,56,57,58].

**Figure 1:**
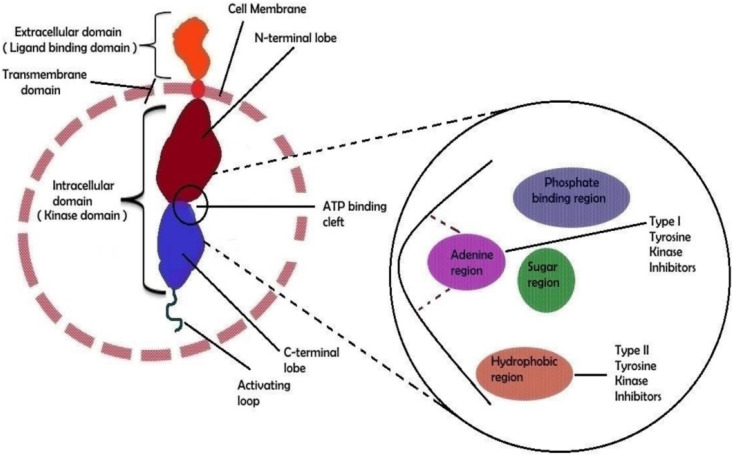
The molecular structural feature of receptor tyrosine kinase (RTK). An RTK’s extracellular domain can bind particular ligands such as growth factors, whereas the intracellular domain is responsible for the kinase’s (auto)phosphorylation. The external and internal domains are separated by the transmembrane region, which is fixed in the cell membrane. The ATP-binding cleft is located between the two lobes of the intracellular domain. A schematic depiction of the ATP binding cleft with its numerous regions is shown on the right side of the image. Type I and type II tyrosine kinase inhibitor binding sites have been shown in a biochemical general structure model. Adapted from [30], Copyright 2021 MDPI.

Through amino acid phosphorylated residues, protein kinases can be divided into three basic groups: serine kinases (serine phosphorylation), threonine kinases (threonine phosphorylation), and tyrosine kinases (tyrosine phosphorylation). In addition, other groups can be found, e.g., histidine protein kinases, but they are not essential for this contribution [30,31,52,53,57,58]. Protein kinases have a typical secondary structure that is divided into 12 subdomains, which form a bilobed catalytic core, to which an ATP molecule is bound in the deep groove (see the schematic structure in Figure 1). The adenine base of the ATP molecule forms hydrogen bonds with a kinase section, the so-called hinge region, which connects N- and C-terminal lobes of the catalytic domain of the protein kinase. The sugar part (ribose) and phosphate residues of the ATP molecule bind in the hydrophilic channel of the protein kinase. All kinases also have an activation loop that is important for the regulation of kinase activity [59]. Pathological protein kinases with mutation (having aberrant structure) or (increased or decreased) expression change affect signaling pathways that provide proliferation advantages to a malignant clone or protect its cells from apoptosis, and thus are the direct reason for uncontrollable cell division [60]. Therefore, protein kinase inhibitors are effective drugs for the treatment of cancers.

Approximately 90 protein-tyrosine kinases (PTKs) have been identified in the human genome, including 58 receptor protein-tyrosine kinases (RTKs), which can be divided into about 20 subfamilies and 32 non-receptor protein-tyrosine kinases (NRTKs), which can be grouped into 10 subfamilies [27,28,30,52,54,57,58,60,61]. RTKs have a transmembrane domain in their molecule and receptors on the cell surface. Different receptor classes belong to this family, e.g., epidermal growth factor receptor (EGFR), vascular endothelial growth factor receptor (VEGFR), platelet derived growth factor receptor (PDGFR), glial cell-line derived neurotrophic factor receptor (GDNFR), insulin-like growth factor 1 receptor (IGF-1R), erythropoietin-producing human hepatocellular receptors (Eph), and discoidin domain receptor (DDR) [27,28,30,52,54,57,58,60,61]. The structure of RTKs is shown in Figure 1. NRTKs have subcellular localization (do not have a transmembrane domain) and can be connected to RTKs, but do not communicate directly with the cell environment. The pathologically changed activity of NRTKs is responsible for cancer growth, proliferation, invasiveness, and metastasizing, for neovascularization of cancer tissue, and for the increased resistance of transformed cells to chemo/radio/immunotherapy. These kinase families include, e.g., Abelson kinase (Abl), sarc kinases (Src), Janus kinase (JAK), and focal adhesion kinase (FAK) [48,57,58].

### 2.2. Tyrosine Kinase Inhibitors

**Figure 2:**
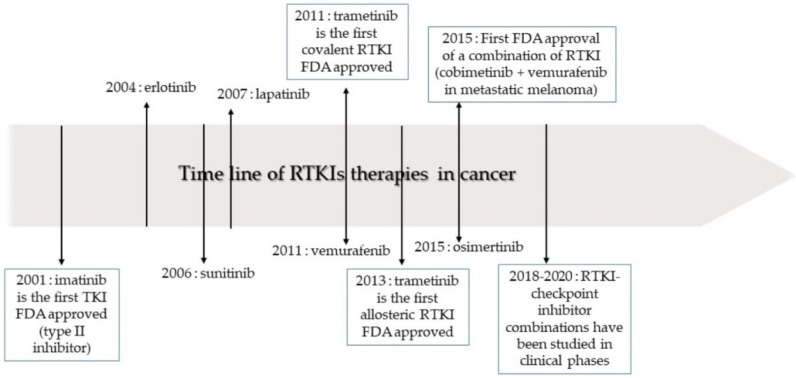
The timeline of receptor tyrosine kinase inhibitor (RTKI) development and approval for the treatment of cancer. Adapted from [31], Copyright 2020 MDPI.

**Figure 3:**
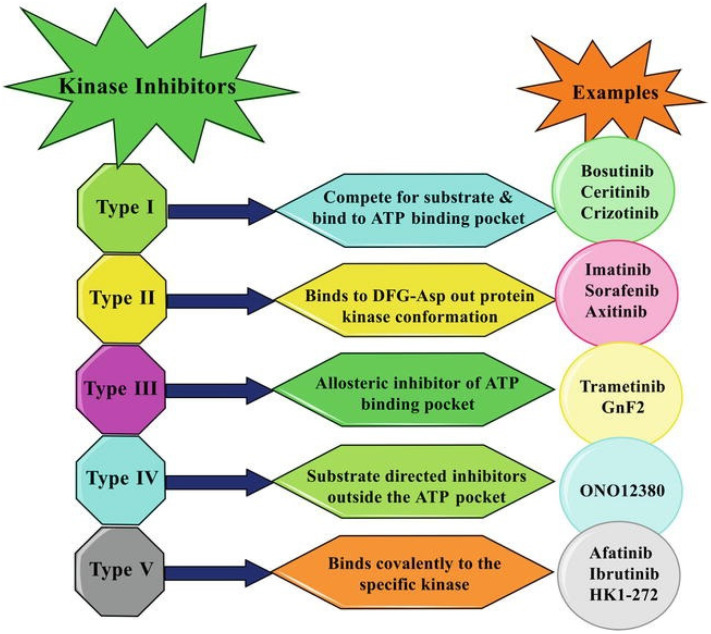
Inhibitory patterns of different kinase inhibitors. Adapted from [63], Copyright 2021 IntechOpen.

**Figure 4:**
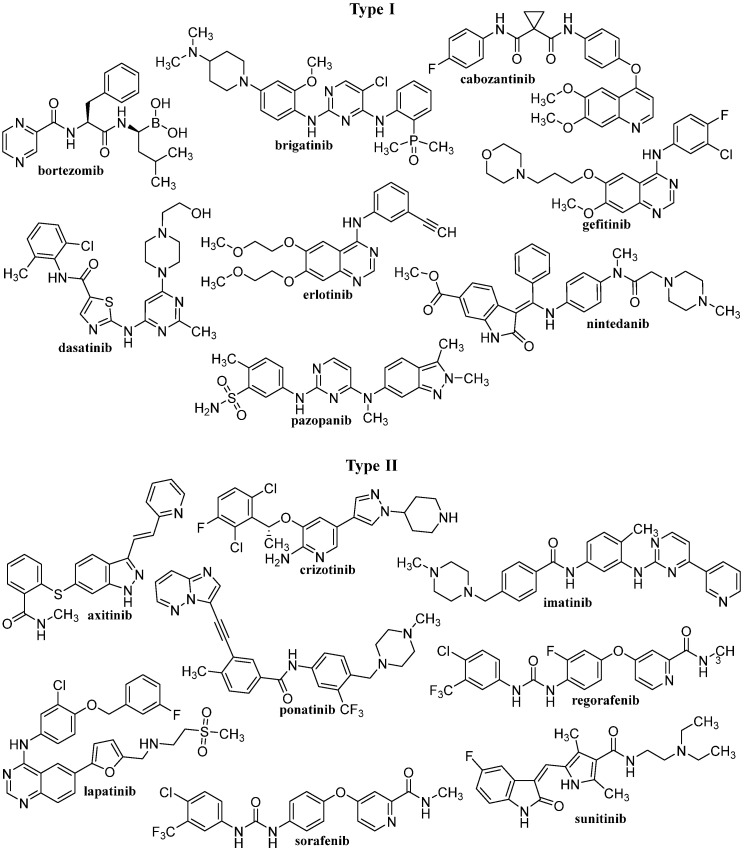
The structures of discussed TKIs of types I and II according to their mechanisms of action.

**Figure 5:**
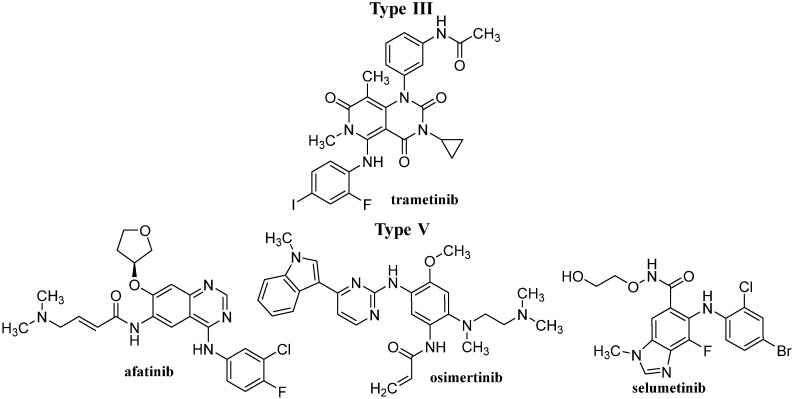
The structures of discussed TKIs of types III and V according to their mechanisms of action.

After IMA success, the group of TKIs increased by several tens of new molecules. The timeline of marketing is presented in Figure 2. A representative list of registered TKIs and TKIs in clinical studies or development is provided in [27,30,31,62]. Generally (irrespective of PTK inhibited), small entities from the TKI group can be classified into several categories according to the mechanism of action, see Figure 3 [31,62,63]. The first group (type I inhibitors) is formed by heterocyclic-based non-covalent ATP-competitive inhibitors that occupy pocket binding purines and serve as a template for side chains for occupation of the hydrophobic region. These inhibitors are basically ATP-binding site competitors and mimic the purine ring of ATP. They bind to the active conformational site and cause the alteration of structural conformation [64]. Type II inhibitors, having phenylalanine in their structure, bind to the site adjacent to the site of ATP kinases in the inactive conformation and stabilize them in their inactive conformation [65]. Type III or allosteric kinase inhibitors bind to the outer catalytic ATP-binding site (remote from the ATP site and the hinge) and are highly selective [66]. Type IV or substrate-directed inhibitors (under development) reversibly attack the substrate binding site, i.e., bind outside the ATP pocket; they are noncompetitive inhibitors and do not compete with ATP [67]. Type V or covalent inhibitors bind irreversibly to the active site of catalytic nucleophile cysteine in the enzyme and thus have reduced off-target side effects [40]. Table 1 contains the characteristics, and Figure 4 and Figure 5 present the formulas of the TKIs discussed below.

Despite unquestionable anticancer therapy benefits of TKIs, some negative aspects should be mentioned. TKIs are intensively metabolized. They are P-glycoprotein substrates, cause its upregulation, and have limited bioavailability. All TKIs and their metabolites are hepatotoxic, and liver damage can be fatal. Besides, they cause hypertension and other cardiovascular/arteriothrombotic adverse events, renal injury, hand–foot skin reaction, persistent diarrhea, nausea, vomiting, and fatigue [62,68,69].

## 3. Nanoformulations of TKIs

**Figure 6:**
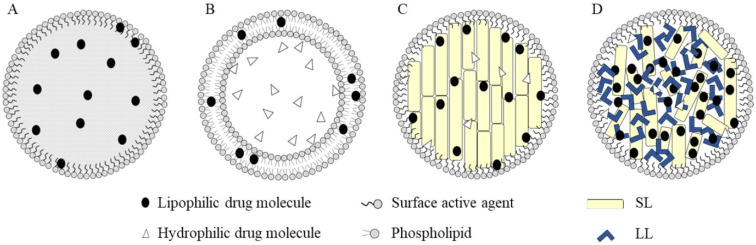
Different types of lipid-based nanoparticles: nanoemulsions (**A**); liposomes (**B**); solid lipid nanoparticles (**C**) and nanostructured lipid carriers (**D**). (SL = solid lipids, LL = liquid lipids) Adapted from [75], Copyright 2020 MDPI.

Classic anticancer chemotherapy with small molecules is limited primarily by the bioavailability of active substance in the target, i.e., afflicted, organ/tissue/cells as compared with accumulation in healthy compartments and a narrow therapeutic window [33,34,70,71,72,73,74]. Drug encapsulation in nanosystems has proved to be an effective strategy to overcome ADME limitations and thus reduce the toxic effect caused by the drug itself [39,75,76,77,78,79,80,81,82,83,84,85,86,87]. Nanocarriers are usually designed so that they can catch in cancer cells and the drug can be released safely and specifically in those cells, which would increase the bioavailability of the drug and minimize the exposure of healthy tissues [78,79,82,83,88]. This can be achieved by passive targeting (increased permeability and retention effect) [89,90] or by active targeting: by covering the nanosystem surface with so-called cancer specific groups (e.g., folate, transferrin, galactosamine), which are specifically recognized by pathologically changed cells being the target [91,92,93,94]. This functionalization of the nanoparticle (NP) surface can help to achieve noteworthy efficacy and decrease the in vivo toxicity of chemotherapeutics. To ensure long circulation in the bloodstream and reduce proteolytic degradation and immunogenicity, the nanosystem surface is standardly covered with polyethylene glycol (PEG) [95]. Nanosystems most commonly get to cells through endocytosis. Controlled drug release from drug delivery nanosystems can happen through simple diffusion from the matrix or hydrolysis caused by pH change or specific enzymes in the target cells. Drug release can be also achieved by external factors, such as magnetism, light, ultrasound, and heat [39,96,97,98]. Emulsions and various lipid vesicular systems are a “gold standard” in drug technology [99]. Therefore, they are frequently used and developed also in the nanoscale age [75,79,80,100,101] (Figure 6). Lipid vesicular systems have been prepared as an alternative to oil in water (*o*/*w*) NEs, where the internal oil phase was replaced with a solid lipid matrix [75,102,103].

It is not surprising that NEs and other lipid-based nanosystems have also come into the sights of technologists reformulating TKIs. For example, therapeutic benefits of bortezomib can be improved using lipid-based nanocarriers, such as liposomes, SLNPs, and microemulsions, which can enhance aqueous solubility, bioavailability, and ensure controlled release of the drug at the site of administration [104]. Targeted delivery of kinase inhibitors using lipid-based delivery systems (liposomes, SLNPs and NLC contributes to the reduction of side effects and ameliorated efficiency of drugs in the target organs. In addition, using combination therapy of TKIs with chemotherapeutic agents or biopharmaceuticals or two TKIs within one formulation may result in synergistic therapeutic effect, reducing side effects and drug resistance in cancer therapy, and is also accompanied by lower costs and better patient compliance [105]. A combination of curcumin with dasatinib using nanoscale drug delivery systems such as liposomes or SLNPs can ameliorate therapeutic efficacy against colon cancer [106]. Anticancer effects of six different kinase inhibitors (crizotinib, erlotinib, foretinib, gefitinib, refametinib, trametinib) encapsulated in a sterically stabilized unilamellar nanocarrier vesicle system containing dipalmitoylphosphatidylcholine, cholesterol, ursolic acid, and PEGylated phospholipid were investigated using HCT116, SW480, H358, HCC827, and A431 cell lines. At combination, the treatment with ursolic acid and kinase inhibitors—mostly synergism—in anticancer effects was observed. Using such co-delivery vesicles with a drug:lipid molar ratio approx. 0.5, the multidrug resistance effect could be likely overcome [107].

### 3.1. Self-Nanoemulsifying Drug Delivery System

**Figure 7:**
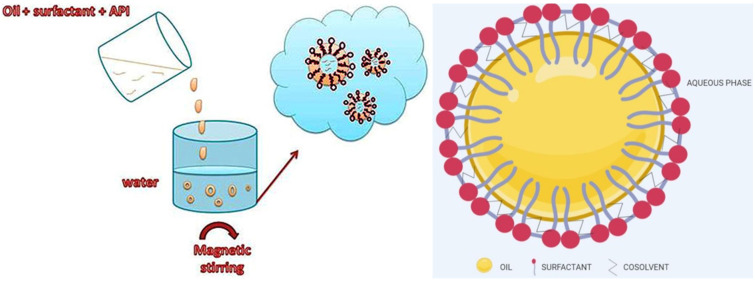
The formulation of SNEDDS and typical structure of SNEDDSs. Adapted from [109], Copyright 2013 OSPC and adapted from [110], Copyright 2020 MDPI.

The self-nanoemulsifying drug delivery system (SNEDDS) is an anhydrous isotopic liquid mixture of oil, surfactant (and usually co-surfactant), drug, co-emulsifier or solubilizer, which spontaneously forms an *o*/*w* NE with a particle size of approximately 200 nm or smaller when diluted with water under gentle stirring. This is an advantageous low-energy emulsification system because emulsification occurs spontaneously [108,109,110,111] (Figure 7). Physicochemical properties, drug solubilization ability, and galenic availability are determined by the selection of SNEDDS components, which can be easily modified. Thus, SNEDDSs can encapsulate not only hydrophobic but also hydrophilic drugs. The encapsulation of drugs in SNEDDSs greatly increases their solubility and overall bioavailability after oral administration. In addition, SNEDDSs prevent drug degradation and improve intestinal permeability. Also important is the fact that liquid SNEDDSs can be converted into solid oral dosage forms (e.g., gelatin capsules filled with liquid SNEDDSs) or solid SNEDDSs [108,109,110,112,113,114,115,116,117].

#### 3.1.1. In Vitro Tested SNEDDS-Based TKI Formulations

SNEDDSs of different composition were prepared with the following TKIs: brigatinib (BG) [118], dasatinib (DAS) [119], IMA [120,121], sorafenib (SOR) [122,123], and sunitinib (SUN) [124]. Their specific composition and particle size is given in Table 2. All these nanoformulations showed a remarkably ameliorated in vitro anticancer effect, enhanced solubility in aqueous medium, and bioavailability compared to bulk drug.

#### 3.1.2. In Vivo Tested SNEDDS-Based TKI Formulations

The flaxseed oil NE encapsulating SOR with particle size 77.46 ± 8.28 nm and zeta potential of –3.4 ± 1.2 mV, which was administered to mice inoculated with Ehrlich ascietes carcinoma cells (EAC^+^) day-by-day via oral gavage with 7 doses (30 mg drug/kg of mice weight) showed smaller tumor volume with increased activity of the lactate dehydrogenase and longer survival (28 ± 2.54 days) compared to free NE and the same dose of drug solubilized in Cremophor^®^ and 95% ethyl alcohol (1:1). Moreover, SOR NE amended the relative liver weight, reduced alanine aminotransferase level, increased the activity of the catalase and reduced damage of the hepatocytes more than the solubilized drug, suggesting the ability of this NE to reduce hepatotoxicity [125]. Similar results were obtained with SOR loaded NE based on carrot seed oil (droplet size: 68.92 ± 10.6 nm) administered to female Swiss Albino mice bearing Ehrlich ascites carcinoma via oral gavage [126]. SUN-loaded SNEDDS with average droplet size 29.5 ± 6.3 nm showed enhanced drug release, ensuring its controlled dissolution as well as cytotoxicity against 4T1 and MCF-7 cancer cells compared to free drug, and at a dose of 50 mg/kg the maximum plasma concentration and the mean area under the plasma concentration time curve were 1.45- and 1.24-fold higher than those observed with SUN suspension [127].

### 3.2. Liposomes

Liposomes are nanoscale drug delivery systems which consist of an amphipathic phospholipid bilayer and an internal aqueous core. These self-assembled lipid-based drug vesicles can form a uni-lamellar or a concentric series of multiple bilayers (multilamellar) surrounding the aqueous compartment, whereby their sizes can range from 30 nm up to 2.5 μm; the thickness of the phospholipid bilayer is 4–5 nm [79,128,129,130,131]. Liposomes have a spherical shape and the core–shell nanostructure enables them to be loaded with both hydrophobic and hydrophilic molecules, whereby hydrophobic drugs are encapsulated in the shell formed by the lipophilic bilayers and hydrophilic drugs are located in the aqueous phase of the core [79,132]. They are suitable for the targeted delivery of drugs, ensuring their controlled release, and can reduce systemic side-effects and improve the therapeutic index of drugs [79,133]. For example, progress in the combinatorial delivery of drugs such as paclitaxel (PTX), topotecan (TPT); SUN, irinotecan, combretastin A-4, and DOX using liposomes, ensuring increased blood circulation, selective drug accumulation at tumor tissues, and stimuli responsiveness, resulting in improved chemotherapeutic effects, was discussed by Jain et al. [134].

#### 3.2.1. In Vitro Tested Liposomal TKI Formulations

Liposomal nanoformulations were prepared and in vitro tested against various human cancer cell lines from the following TKIs: ERL [135], IMA [136,137,138], SOR [139,140] and afatinib (AFT) [141]. Their specific composition and particle size are given in Table 3. Liposomal formlations demonstrated long-term stability, sustained release, enhanced cellular uptake, and anticancer effect in comparison with bulk drugs. In the case of the combination of IMA with classical antitumor drugs such as paclitaxel (PTX) [137] and tamoxifen [138], a synergistic effect was observed, resulting in further strengthening of the effect. Also, a liposomal nanoformulation consisting of egg phosphatidylcholine and cholesterol for co-delivery of lapatinib (LPT) and PTX, showing pronounced inhibitory activity against P-glycoprotein which are responsible for efflux pump mediated multidrug resistance, was prepared by Ravar et al. [142]. This liposomal formulation with mean particle size of 235 ± 12 nm and EE of 52% and 68% for LPT and PTX, respectively, released after 40 h 93% PTX and 71% LPT and exhibited improved cytotoxicity to 4T1 mouse breast cancer cells compared with the binary mixture of free drugs. On the other hand, Patel et al. [143] prepared nanoliposomes consisting of Phospholipon^®^ 90H and cholesterol suitable for inhalation. These SOR tosylate loaded liposomal dry powder showed optimized liposomes with a particle size of 111.15 ± 1.03 nm, zeta potential of 29.87 ± 0.56 mV, 93.13 ± 1.11% EE, and low density and good flowability. Based on the in vitro deposition fine particle fraction of 83.7 ± 0.09%, mean mass aerodynamic diameter 3.15 ± 0.2 μm, geometric standard deviation 1.78 ± 0.15 μm and dispersibility of 85 ± 0.1% was estimated and biphasic release pattern was observed with burst release in the first 6 h and following sustained release up to 72 h, suggesting the potential of SOR tosylate for NSCLC treatment. A very remarkable but comprehensive study was recently published by Salmaso et al. [144]. A synthesized prodrug Pro962 of TKI TK962 was loaded into liposomes consisting of egg phosphatidylcholine, cholesterol, and 1,2-dihexadecanoyl-sn-glycero-3-phosphoethanolamine with a size in the range of 120–190 nm showing pH-controlled release in the tumor, whereby a cholesterol moiety was linked to TK962 through pH-sensitive hydrazone bond and anchored to the liposome phospholipid bilayer, ensuring prevention for Pro962 leakage from liposomes. Because in this formulation Pro962 was de facto associated with the vesicles, the drug release was restricted under blood-mimicking conditions (in contrast to TK962-loaded conventional liposomes, which showed fast release of the drug) and approximately half of the drug was released at pH 4 and pH 5 in 2 h. The Pro962-loaded liposomes exhibited increased cytotoxicity compared to unencapsulated TK962 in both 2D and 3D models (BxPC3 and PSN-1 pancreatic adenocarcinoma cell lines and A431 human squamous cervical carcinoma cell line) and the treatment of human, mouse, and rat microsomes showed that they attenuated the metabolic reactions and protected Pro962 from catabolism [144].

#### 3.2.2. In Vivo Tested Liposomal TKI Formulations

Liposomes encapsulating a multi-receptor TKI cabozantinib (CBZ) showed higher cytotoxicity than free CBZ and exhibited sustained inhibition of phosphorylation of Met, protein kinase B (AKT), and mitogen-activated protein kinase (MAPK) pathways in renal cell carcinoma (RCC) cells. The liposomal formulation exhibited sustained inhibition of tumor growth and was more efficient than free CBZ in a RCC tumor xenograft model due to the improved accumulation of liposomes in the tumor [145]. Nanoformulation consisting of nanoliposomes doped with a photoactivable benzoporphyrin derivative XL184 (activated by NIR irradiation using a 690 nm) as a chromophore in the lipid bilayer—and containing CBZ NPs, whereby the multikinase inhibitor was encapsulated inside nanoliposomes—were prepared by Spring et al. [146]. Nanoliposomes were prepared using the following ingredients: 1,2-dioleoyl-3-trimethylammonium-propane, 1,2-dipalmitoyl-sn-glycero-3-phosphocholine, 1,2-distearoyl-sn-glycero-3-phosphoethanol- amine-*N*-[methoxy(polyethylene glycol)-2000], poly(d,l-lactic-co-glycolic acid)–polyethylene glycol, and had an average particle size of 50 nm. The system was tested in vitro on human pancreas adenocarcinoma ascites AsPC1 cells, and in vivo efficacy was verified in two mouse models of pancreatic cancer. Tumor irradiation using near-infrared radiation (NIR) following intravenous (i.v.) administration of these photoactivable multi-inhibitor nanoliposomes induced photodynamic impairment of tumor cells and microvessels, resulting in CBZ release inside the tumor. Even administration of a single dose of this formulation was able to extend tumor reduction in two mouse models and restrain metastatic escape in an orthotopic pancreatic tumor model, suggesting that using the prepared nanoliposomes, spatiotemporal control of drug release can be achieved and toxic impact of systemic drug treatment can be reduced. Asolectin-based liposomal formulation with encapsulated erlotinib (ERL), showing mean particle size 121 ± 10.7 nm, zeta potential of –33.7 ± 2.30 mV and EE of 82.60% exhibited improved effectiveness against PANC-1 cells in vitro compared to free drug (IC_50_: 1.1 ± 0.1 μg/mL vs. 2.0 ± 0.3 μg/mL), caused cell apoptosis and arrested the G_0_/G_1_ phase of cell cycle, whereby a hemolysis study showed that this formulation was safer than the drug solution [147].

Li et al. [148] fabricated multifunctional liposomes with anti-EGFR aptamer-conjugated chitosan (CS) able to deliver encapsulated ERL and perfluorooctylbromide (PFOB) to EGFR-overexpressing non-small cell lung cancer (NSCLC), whereby the entrapped PFOB promoted the uptake of liposomes in either normoxia or hypoxic condition. This liposomal nanoformulation can contribute to overcoming hypoxia-evoked erotinib resistance both in vitro and in vivo. An anionic liposome nanosystem consisting of lecithin, phosphatidylserine showing “sandwich” structure with encapsulated TPT in the lipid hydrophilic layer, indocyanine green sensitizer loaded into the hydrophobic layer and positively charged ERL adsorbed to the outermost layer of the microenvironment entered the tumor through normalization of blood vessels after the action of ERL, whereby using ultrasound ameliorated the vascular permeability enabling penetration of NPs into blood vessels and reach tumor cells; in addition, TPT down-regulated the expression of hypoxia-inducible factor (HIF)-1α, which led to prolongation of the vascular normalization time. The obtained in vivo results from mouse model of breast cancer 4T1 confirmed improvement of the tumor environment at treatment with liposomal nanoformulation and increased anticancer effectiveness due to combination of vascular normalization combined with sonodynamic therapy and chemotherapy [149]. Ultrasound-triggered and magnetic-targeted nanobubble system for dual delivery of pemetrexed (suitable for treatment of NSCLC) and pazopanib were prepared by attaching of peptide drug conjugates to amine-modified Fe_3_O_4_ and subsequently the formed NPs were encapsulated into liposomes, which were extruded, and the nanobubble system showing sizes 491.1 ± 130.2 and 275.8 ± 117.8 nm was fabricated. From the injected carrier system, 80.22% accumulated into the tumor area; accumulation of nanobubbles responded to magnetic field application and focused acoustic pressure resulted in the disruption of nanobubbles, leading to targeted drug delivery [150].

Axitinib (AXT)-loaded spherical polypeptide-coated hybrid liposomal core-shell NPs prepared using 1,2-dipalmitoyl-sn-glycero-3-phosphocholine (DPPC), cholesterol, and dimethyldioctadecylammonium bromide (DDAB) via a thin-film hydration technique, which were coated with PEG-b-poly(aspartic acid) (PAsp) through electrostatic interactions (P-LNP/AXT), showed considerably slower drug release at pH 7.4 (ca. 8%) compared with pH 5.4 (22%) within 48 h. This can be associated with increased swelling at lower pH or by the modified fluidity of the liposomal bilayer membrane, whereby the release of AXT from the core is controlled by the PEG-b-PAsp layer. P-LNP/AXT showed cytotoxicity to SCC7, BT-474, and SH-SY5Y cells, although at a dose of 100 μM, the viabilities of cells treated with free drug were lower than those treated with P-LNP/AXT due to the sustained release of AXT from the nanoformulation. It can be supposed that P-LNP/AXT can evade the reticuloendothelial system because it is not pronouncedly internalized by the mouse macrophage cell line RAW 264.7. Treatment with P-LNP/AXT considerably increased the level of expression of hydroxy-HIF-1α and remarkably inhibited the growth of tumors in mice compared to the control group; based on the increased levels of caspase-3 and poly (ADP-ribose) polymerase and reduced levels of platelet/endothelial cell adhesion molecule 1 (PECAM1, also known as CD31) and Ki-67 protein in tumor cells apoptosis of cancer cells and inhibition of angiogenesis within the tumor was proved [151]. Folate receptor (FR)-targeting IMA-loaded liposomes with average particle size 143.5 nm, zeta potential of −15.97 mV and EE > 90% exhibited >25% drug release in phosphate-buffered saline (PBS) at pH 5.5 within 72 h, while in PBS at pH = 7.4 the observed release did not achieve 5%. The cytotoxicity of FR-targeted liposomes containing IMA against HeLa cells (IC_50_: 150 μM) was 6-fold lower than that of non-targeted ones (910 μM) and also increased apoptosis of HeLa cells in vitro. In addition, FR-targeted IMA liposomes enhanced HeLa cell apoptosis in vitro compared to the non-targeted IMA liposomes. The improvement of long circulation properties in Kunming mice was observed at treatment with both targeted and non-targeted liposomes [152]. Magnetic nanocomposite based on ZnFe_2_O_4_-IMA-liposomes suitable for targeted IMA delivery exhibited stimulated drug release under alternative magnetic field in vitro due to motions of NPs in liposomal nanocomposite caused by modified permeability of the bilayer, and in the in vivo experiment more efficient accumulation of magnetically controlled liposomes in the targeted sites was observed [153]. Doxorubicin (DOX) and IMA co-loaded into pH-sensitive folate receptor targeted liposomes with a particle size of about 100 nm preserved stability in blood circulation and exhibited fast release of both drugs in tumor acidic microenvironment, considerably improved anticancer effects both in vitro and in vivo, and were able to overcome DOX-associated chemoresistance via inhibition ABC transporter function by IMA [154].

SOR-loaded glycol chitosan-coated liposome (GC-SF-Lip), and Eudragit^®^ S100-glycol-chitosan coated liposomes (SGC-SF-Lip) were stable at acidic and neutral pHs and prevented drug leaching in contrast to their uncoated counterparts, which were unstable at pH 1.2. Besides both of the above-mentioned coated liposomal formulations, the double coated SGC-SF-Lip formulation also exhibited higher cellular uptake in Caco-2 cells than the drug solution, although SGC-SF-Lip showing comparable cellular uptake to GC-SF-Lip at pH 7.4, due to removal of the Eudragit^®^ S100 coating layer, exhibited lower cellular uptake than GC-SF-Lip at pH 6.5, suggesting lower toxicity of SGC-SF-Lip in acidic environment. Considerably improved effectiveness of SGC-SF-Lip after oral administration to rats was reflected in observed maximum serum concentration of drug (C_max_) and area under the curve (AUC), which were fourfold higher compared to free drug [155]. SOR and indocyanine green, a photodynamic therapy agent, co-loaded in NIR fluorescence imaging-guided photothermally sensitive nanoliposomes showed improved biocompatibility, biotoxicity, and anti-tumor effects in Hep3B tumor-bearing xenograft nude mice compared to free SOR can be considered as promising nanoformulation for advanced hepatocellular carcinoma therapy [156].

SUN-loaded liposomes decorated with cyclo-aminoprolineRGD units (cAmpRGD, selective ligands for integrin alpha(v)beta(3) (αVβ3), which targets the liposomes to the integrin αVβ3-overexpressing cells and supports their active cell internalization, resulting in the accumulation of SUN, were in an in vivo study found to inhibit angiogenesis more than the free drug or untargeted drug-loaded liposomes. The prepared SUN-loaded targeted liposomes enable reduction of the administered drug, along with a decline of adverse side-effects, and thus can ensure prolongation of the antiangiogenic therapy [157]. Targeted liposomes (90–100 nm) prepared using d-α-tocopheryl PEG 1000 succinate (TPGS(1000))–triphenylphosphine conjugate with encapsulated SUN or vinorelbine, which were tested against highly invasive breast cancer MDA-MB-435S cells, showed prolonged blood circulation, an enhanced permeability retention effect in cancer tissue of xenograft tumor BALB/C nude mouse model, and a mitochondrial targeting effect. They accumulated in the mitochondria of MDA-MB-435S cells or vasculogenic mimicry (VM) channel-forming cancer cells, causing acute cytotoxic damage and apoptosis, whereby caspase 9 and caspase 3 were activated and VM channel-forming indicators (MMP-9, EphA2, VE-Cadherin, FAK and HIF-1α) downregulated. Consequently, combination of targeted SUN liposomes and targeted vinorelbine liposomes have potential to be used in effective treating of invasive breast cancer along with preventing relapse originating from VM channels [158]. NIR-activated IR780-loaded liposomes with encapsulated SUN, in which IR780 is situated in the liposome phospholipid bilayer, and disruption of the bilayer by laser irradiation results in the release of SUN, which will be activated remotely at the tumor site and subsequently targets multiple VEGF receptors on the tumor endothelial cell surface, leading to the inhibition of angiogenesis, were designed by Yang et al. [159]. In addition, IR780-loaded liposomes kill the cancer cells by photothermal therapy. The advantage of this nanoformulation is the controlled release of the encapsulated drug inhibiting angiogenesis along with photothermal therapy. This nanoformulation exhibited enhanced anti-tumor and anti-angiogenic effects in vitro and in vivo on a syngeneic female BALB/c mouse tumor model which was established with the 4 T1 cell line [159].

Encapsulation of the FGFR inhibitors ponatinib, PD173074, and nintedanib into liposomes consisting of 1,2-distearoyl-sn-glycero-3-phosphocholine, cholesterol, 1,2-distearoyl-sn-glycero-3-phosphoethanolamine-*N*-[methoxy(polyethylene glycol)-2000)] with a particle size from 96 to 122 nm, reduced short-term (up to 72 h) cytotoxicity in FGFR1-driven lung cancer cell lines DMS114, NCI-H520 and NCI-H1703 at higher concentrations compared to free drugs, although in long-term clonogenic experiments the drug-loaded liposomes showed comparable or higher efficiency than free drugs. In contrast to free ponatinib, using its liposomal formulation resulted in considerable tumor growth inhibition (up to 60.4%) in an FGFR inhibitor sensitive murine osteosarcoma transplantation model (K7M2), along with significantly reduced side effects [160].

### 3.3. Solid Lipid Nanoparticles

SLNPs consist of matrix materials, i.e., lipids such as triglycerides, fatty acids, cholesterol, waxes, partial glycerides, fats, and the surface stabilizers, including phospholipids, bile salts, soyabean lecithin, egg lecithin, phosphatidylcholine, poloxamers, and polysorbates, whereby the solid lipid core is enclosed into a lipid monolayer [161,162,163,164]. The size of SLNPs ranges from 50 nm to 1000 nm, they can cross different physiological barriers and limit mobility of drug molecules in a solid lipid matrix [161], whereby hydrophobic compounds are solubilized within the central solid-lipid core of SLNPs in the presence of suitable surfactants [162]. Thanks to their composition, SLNPs have low toxicity, good biodegradability and high physical stability, which is also reflected in the increased stability of encapsulated drugs. They provide protection of drugs before the first pass effect, increase the lymphatic transport of drugs, and by changing the lipid components, tunable properties can be achieved for the controlled release of hydrophilic and lipophilic drugs [79,130,165,166]. A review paper discussing targeted delivery of anticancer TKIs encapsulated in SLNPs was presented by Satapathy and Patro [167]. Lipid–polymer hybrid nanoparticles (LPH NPs) are core–shell nanostructures, where a polymer core remains enveloped by a lipid layer and the outer surface can be functionalized for active targeting of anticancer therapy, used as a diagnostic imaging agent, etc. [168,169,170].

#### 3.3.1. SLN-Based TKI Formulations Tested In Vitro

SLNPs were prepared and in vitro tested especially against lung, breast, and liver cancer cell lines from the following TKIs: BG [171], gefitinib [172], ERL [173,174,175,176], IMA [177], SOR [178,179,180,181,182,183,184], SUN [185,186], and AFT [187,188,189]. SLNP formulations demonstrated good stability, sustained release, targeting, enhanced cellular uptake, and the induction of apoptosis, resulting in higher anticancer effect in comparison with bulk drugs. Some formulations are very interesting; on the one hand, they combine TKIs with other clinically used antineoplastics (PTX, cisplatin) [176,183,189], while on the other hand, their surface is covered with specifically targeting peptides (SP94, FD7/CCD) [181,188], or they enhance the specific distribution also with the addition of magnetic particles [184].

Vivek and Jose designed SLNPs fabricated using IMA mesylate, Compritol^®^ 888 ATO and Pluronic^®^ F6 showing a mean particle size of 190 nm and an EE of 62.5% for specific targeting to mesenteric lymph nodes, which was verified using various laboratory methods [190]. On the other hand, the time-dependent uptake and cytotoxicity of IMA lipid nanocapsules with a mean particle size of 38.96 ± 0.84 nm, zeta potential of −21.5 ± 0.61 mV, and 99.17% EE were tested on B16F10 murine melanoma cells [191]. Ponatinib has also been tested in vitro in murine cancer cell lines. Ponatinib-encapsulating leukosomes, i.e., lipid NPs enriched with membrane proteins derived from activated leukocytes on the surface, exhibited cytotoxicity against murine osteosarcoma cell lines F420 and RF379 in a dose-dependent manner [192]. The specific composition, particle size, and benefits of other formulations tested in vitro on human cancer cell lines are shown in Table 4.

#### 3.3.2. In Vivo Tested SLN-Based TKI Formulations

Pazopanib-loaded SLNPs with a particle size of 210.03 ± 7.68 nm, EE of 79.05 ± 2.55% and zeta potential of −18.29 ± 1.89 mV showed improved cellular uptake and powerful cytotoxicity to A549 lung cancer cells in vitro associated with apoptotic mechanism and inhibited tyrosine kinase. The formulation was characterized with considerably enhanced bioavailability and sustained-release pattern, releasing 92.67 ± 4.68% of drug within 24 h, as well as excellent lung targeting as was verified in Wistar albino rats [193].

ERL NPs prepared by nanoparticulation using fat and supercritical fluid with a mean size of 250 nm strongly inhibited epidermal growth factor (EGF) signaling and suppressed proliferation of A549, a human NSCLC cells; in vivo study with A549 xenografts in BALB/c nude mice showed that the NPs not only regressed the tumor more efficiently than Tarceva^®^, but also exhibited more efficient inhibition of lung metastasis than Tarceva^®^; these NPs showed 5.5-fold higher bioavailability of ERL than Tarceva^®^ [194]. In an in vivo study with Sprague Dawley rats, the comparison of free ERL with ERL hydrochloride-loaded SLNPs with mean particle size 177 nm and zeta potential of –33 mV showed that the SLNPs exhibited a 2.12-fold increase in the oral bioavailability and a reduction of variability in the AUC from 2.5 to 1.4 from fed to fasted state [195]. PEGylated core–shell type polypeptide lipid nanocapsules (LNCs) with encapsulated ERL, showing mean size of 200 nm and zeta potential of −20 mV were able to control drug release from the nanocapsules, showing faster drug release under acidic conditions and dose-dependent cytotoxicity in NCl-H358 and HCC-827 lung cancer cells. This nanofomulation also effectively suppressed tumor growth in a xenograft tumor model compared to free ERL and control, achieving 5- and 2-fold smaller tumor volume in treated mice compared to control nanocapsules and free drug, suggesting its potential to be used for lung cancer therapy [196]. Poly(acrylic acid)-cystamine-oleic acid-modified ERL-loaded NPs (PAA-ERL-NPs) with mean size 170 nm, zeta potential of −32 mV and 85% EE showed sustained cumulative drug release for 72 h and exhibited higher in vitro cytotoxicity against A549 and NCI-H460 cells (IC_50_: 3.3 ± 0.3 and 4.6 ± 0.5 μM) than non-functionalized ERL-loaded SLNPs (IC_50_: 9.5 ± 0.7 and 17.2 ± 1.1 μM) and ERL solution (IC_50_: 36.8 ± 2.3 and 46.5 ± 3.1 μM). Similarly, also in an in vivo experiment using a xenograft nude mouse model with human lung cancer cells the tumor inhibition rate after 21 days decreased as follows: PAA-ERL-NPs (84.5%) > ERL NPs (68.7%) > ERL (38.1%). Hence, redox-responsive poly(acrylic acid) ligands showing pH sensitivity stimulated NPs to deliver drug into the tumor cells and the structure of NPs enabling delayed drug release ensured a long-lasting drug delivery in tumor tissues [197]. 

SLNPs fabricated by Ganthala et al. [198] using CS-maleic anhydride-TPGS polymer, which were loaded with ERL and quercetin, showed average particle size of 87.3 ± 0.78 nm, zeta potential of +13.4 ± 1.12 mV and 77% and 71.4% EE for ERL and quercetin, respectively. These SLNPs reduced the expression of P-glycoprotein and nuclear EGFR, showed pH dependent sustained release till 72, with higher release at acidic pH, and enhanced the uptake of both drugs, achieving 55.80% apoptotic cell percentage in ERL resistant A549/ER cells. In an in vivo study increased uptake of SLNPs containing both drugs in lung tissue was associated with the enhanced permeability and retention (EPR) effect, pH-responsive properties, diminished P-glycoprotein efflux, and activated antioxidant defense in normal cells by quercetin. Remarkable inhibition of the expression of nuclear EGFR/PI3K/AKT protein in ERL resistant A549/ER cells in vitro and in C57BL6 mice with metastatic lung tumors tissues in vivo was observed as well. This nanoformulation can be used in targeted therapy of NSCLC with minimum side effects [198]. He et al. [199] designed a pH-sensitive lipid bilayer (HHG_2_C_18_-L) using zwitterionic oligopeptide lipid, 1,5-dioctadecyl-l-glutamyl-2-histidyl-hexahydrobenzoic acid, for coating NH_2_-functionalized mesoporous SiO_2_ NPs and incorporated ERL and DOX in this nanoformulation.

ERL, which was sequestered in the exterior lipid bilayer, released faster than DOX during the cellular transport. At tumor intracellular pH the HHG_2_C_18_-L became more positive resulting in increased gradual release of both drugs, and remarkable synergistic effects in antiproliferation and apoptosis of A549 human cancer cells was observed in vitro. In an in vivo study, the nanoformulation with incorporated ERL and DOX exhibited pronounced accumulation of nanoformulation and powerful inhibition of tumor growth in Lewis lung carcinoma tumor bearing mice and prolongation of survival period without any sign of systemic toxicity was observed. pH sensitive LPH NPs co-encapsulating ERL and bevacizumab (BEV) (HA-ERL/BEV-LPH NPs), which were functionalized with HA containing pH sensitive adipic acid dihydrazide (HA-ERL/BEV-LPH NPs), with sizes approx. 100–120 nm and negative zeta potentials exhibited faster drug release at pH 5.5 than at pH 7.4, in contrast to non-functionalized LPH NPs showing comparable drug release at both pH. HA-ERL/BEV-LPH NPs exhibited higher in vitro cytotoxicity on A549 and H1975 cells compared to free drugs and single drug loaded formulation; HA-ERL-LPH NPs also achieved higher (>70%) uptake into A549 cells compared to ERL/BEV-LPH NPs (52.3%), and in an in vivo experiment using the NSCLC mice model, they reduced tumor volume to greater extent and showed higher tumor inhibition rate in tested animals than HA-ERL-LPH NPs and HA-BEV-LPH NPs. Using HA-ERL/BEV-LPH NPs maximum plasma ERL concentration, life period and tumor tissue accumulation of ERL were 21.6 μg/mL, 7.57 h, and 25.3 μg/mL [200].

Combined NPs, in which celastrol (CST) was loaded in the mesoporous SiO_2_ NPs and AXT was loaded in the coating consisting of PEGylated lipidic bilayers, strongly inhibited angiogenesis and mitochondrial function. These combine NPs were effectively internalized in SCC-7, BT-474, and SH-SY5Y cancer cells, greatly reduced HIF-1α expression under hypoxic conditions in tested cancer cells and induced synergistic cancer cell apoptosis. In such combined NPs AXT controls VEGFRs and CST acts on target cell mitochondria, what results in synergistic effect. Besides strong tumor inhibition in vivo using the tumor xenograft mouse model, increased caspase-3 and poly (ADP-ribose) polymerase and reduced CD31 and Ki-67 expression was observed, suggesting tumor apoptosis via mitochondrial and antiangiogenic effects [201]. 

SLNPs incorporating LPT with mean particle size 88.6 nm and a zeta potential of 20 mV were efficiently taken up into C6 glioma cells, and in vivo showed a relative higher AUC compared to Tykerb^®^ and LPT suspension and a half lethal dose > 250 mg/kg [202]. NPs consisting of a lipid corona and LPT and albumin as a core were efficiently taken up by BT-474 cells and induced apoptosis; in vivo they were passively distributed into a tumor via the EPR effect and showed improved antitumor activity in breast cancer cells [203]. NPs containing LPT bound to albumin as a core, and egg yolk lecithin forming a lipid corona with mean particle size 62.1 nm and zeta potential of 22.80 mV applied at a dose 20 μg/mL induced considerable cell arrest at G_0_/G_1_ phase compared with the same concentration of drug suspension; after intravenous administration to mice bearing BT-474 xenograft they targeted and accumulated in tumors and co localized with HER2 and SPRAC (secreted protein, acidic and rich in cysteine) [204]. LPH NPs consisting of poly[lactide-co-glycolide]-d-α-tocopheryl polyethylene glycol 1000 succinate enveloped by a PEGylated DSPE lipid layer, which were loaded with LPT, showed accelerated release at pH 5.5, superb internalization and inhibition of proliferation of MCF-7 cancer cells as well as higher apoptosis of cancer cells than the free drug, Moreover, these LPH NPs were able remarkably enhance the blood circulation time of drug due to reduced uptake by a reticuloendothelial system what supported preferential accumulation of drug in the tumor tissues. The LPH NPs showed antitumor activity also in vivo what was reflected in reduced tumor cell proliferation and enhanced apoptosis in cancerous mice [205]. In an in vivo study it was shown that combine treatment with LNCs encapsulating SN-38 (an antineoplastic drug) and LNCs encapsulating regorafenib reduced CT26 murine colorectal tumor growth and prolonged median survival time. Encapsulation of drugs into nanocapsules also considerably reduced the haemolysis [206].

SOR encapsulated in LNCs showing a size 54 ± 1 nm and EE > 90% inhibited in vitro angiogenesis and reduced human U87MG GB cell viability; their intratumoral administration to nude mice bearing an orthotopic U87MG human GB xenograft reduced the amounts of proliferating cells in the tumor relative to control groups, whereby the LNCs were more efficient compared to free drug and were able to induce early tumor vascular normalization via increasing tumor blood flow and reduction of tumor vessel area [207]. SOR-loaded lipid-coated nanodiamond system increased drug oral bioavailability 7.64-fold, enhanced drug concentration in gastric tumor tissue 14.95-folds and also strongly suppressed tumor growth in tumor xenograft model; in addition, the metastasis of liver and kidney were greatly suppressed as well [208]. SOR-loaded lipid-based nanosuspensions consisting of particles showing a size 164.5 nm and zeta potential of −11.0 mV exhibited higher in vitro cytotoxicity against HepG2 and Bel-7402 cancer cells than free SOR, and in vivo, using H22-bearing liver cancer xenograft murine model, exhibited improved antitumor efficacy reflected in reduced tumor volume and higher accumulation in the tumor tissue compared to free drug administered per os or intravenously [209]. HA/lipid hybrid NPs encapsulating SOR released the drug in response to hyaluronidase and exhibited enhanced SOR accumulation at tumor site in vivo, resulting in ameliorated antitumor efficacy [210].

Lipid prodrug containing HA and cisplatin prepared using PEG as a linker and SOR incorporated into SLNPs with mean particle size 173.2 ± 5.9 nm, and zeta potential of −21.5 ± 3.2 mV showed antiproliferative activity against MKN28 and SGC7901 human gastric cancer cells, and in an in vivo study reduced tumor volume from 1532.5 ± 41.3 mm^3^ to 259.6 ± 16.3 mm^3^ within 21 days without body weight loss of animals, while administration of free drug resulted in body weight loss from 15–20 g within 3 weeks [211]. *N*-Acetylgalactosamine modified and pH sensitive LNPs co-encapsulating DOX and SOR showing synergistic effects of both drugs in antitumor activity on human hepatic carcinoma (HepG2) cells in vitro and in antihepatic carcinoma mice model in vivo was reported previously by Duan et al. [212]. Optimized SLNPs modified with tumor-targeting peptide iRGD showing a shell-core structure with incorporated DOX and SOR exhibited cytotoxicity, pro-apoptotic impact and increased internalization rate of HepG2 cancer cells in synergistic manner; in vivo they prolonged circulation and bioavailability of drugs and considerably increased antitumor effectiveness in HCC xenograft mouse models [213].

Low-density lipoprotein (LDL)-mimetic lipid nanoformulations composed of apolipoprotein B to improve efficiency for LDL receptor-over expressed liver tumors, which were used for co-delivery of SOR and dihydroartemisinin, reduced the viability of HepG2 cells and ensured 3-fold higher SubG1 percentage of cells compared to the treatments with a single drug; they also strongly delayed tumor growth in vivo, achieving considerably lower proliferation index (22.1 ± 5.6%) in xenograft tumor model compared to control (86.2 ± 6.9%), pure SOR (75.4 ± 4.89%) or pure dihydroartemisinin (69.4 ± 6.9%) [214]. Arginine-glycine-aspartic acid (RGD) modified lipid-coated PLGA NPs co-loaded with SOR and quercetin suppressed viability of HCC cells more than the single drug formulated into NPs or their solutions and showed remarkable tumor growth inhibition in vivo [215]. Cellular uptake, cytotoxicity, and gene-silencing studies in HepG2, and Hepa 1–6 cells supported the selectivity to HCCs compared to HeLa cancer cells and FL83B normal cells, whereby using pH-sensitive lipid, YSK05, resulted in enhanced cytotoxic and gene knockdown efficiencies and restricted extracellular drug release. Anti-GPC3 antibody tagged cationic switchable lipid-based NPs encapsulating SOR and anti-miRNA27a showed pH-responsive release of SOR, specific affinity towards the GPC3-overexpressed HepG2 cancer cells and exhibited reduction of viable cancer cells along with considerable increase of apoptosis compared to free SOR, which was associated with the presence of anti-microRNA27a considerably increasing the protein expression of forkhead box protein O1 (FOXO1), and peroxisome proliferator-activated receptor gamma (PPAR-γ), which are key components involved in the proliferation and apoptosis of tumor cells. The lipid-based NPs also efficiently suppressed tumor burden in vivo, and in liver cancer xenograft model reduced tumor burden, and caused apoptosis without causing toxicity to animals [216].

SLNPs co-encapsulating superparamagnetic iron oxide nanoparticles (SPIONs) and SOR showing mean size of <200 nm retained their superparamagnetic behavior, showed enhanced accumulation inside the HepG-2 liver cancer cells in vitro under application of an external magnetic field and over 72 h exhibited more controlled and sustained release than free SOR. In an in vivo experiment, an under-skin implantation of separated two magnets above the mouse liver ensured better targeting and improved accumulation of SPIONs in the mouse liver along with ameliorated targeting, which was greatly affected by magnetic field topography [217]. A nanocomposite consisting of superparamagnetic iron oxide nanocubes and pH responsive synthetic peptides with lipid tails (octadecylamine p(API-L-Asp)(10)) able to release encapsulated SOR at acidic pH was designed by Park et al. [218] for magnetic resonance imaging (MRI)-monitored transcatheter delivery of SOR. In an in vivo study using an orthotopic HCC rat model and transcatheter hepatic intra-arterial injection of the nanocomposite, the drug was effectively delivered, and what was confirmed with MRI and considerable suppression of tumor growth in a rodent HCC model was observed. Based on these results it can be stated that such nanocomposites containing SOR have potential to be used for liver-directed intra-arterial treatment of unresectable HCC.

Transferrin-modified redox-sensitive SLNPs loaded with AFT with average particle size 103.5 ± 4.1 nm and zeta potential of −21.2 ± 2.4 mV showed glutathione (GSH)-triggered drug release behavior and higher in vitro cytotoxicity to H1975, and PC-9 cells (NSCLC cell lines) in hypoxic conditions than unmodified AFT–SLNPs and bulk AFT, and also in vivo, in Balb/c-nude mice with subcutaneously injected H1975 cells, they inhibited the tumor volume from 919 mm^3^ to 212 mm^3^ [219]. Investigation of inhaled microspheres systems prepared by AFT loading in stearic acid-based SLNPs, which were subsequently loaded together with PTX into PLGA-based porous microspheres, showed that AFT and PTX exhibited a synergistic effect and high efficiency against drug-resistant NSCLC cells. High lung concentration of drugs for 96 h was maintained in Sprague-Dawley rats, while in other tissues it was low. Thus, such drug combination therapy can be effective for drug-resistant lung cancer and enables to overcome resistance that often occurs after 9–13 months of EGFR TKIs administration in NSCLC [220].

### 3.4. Nanostructured Lipid Carriers

NLCs are the second generation of the SLNPs system, in which a portion of solid lipid is replaced by oil, and this unstructured, less ordered lipid matrix can ameliorate the loading efficiency of drugs and hinder leaching and oxidation of drugs during storage, whereby at room and body temperature the NLCs exist as a solid matrix of lipids [164,221]. NLCs are suitable for controlled drug delivery [222], improve the solubility of hydrophobic drugs, and via their surface modification site specific targeting of drugs can be achieved and drug resistance in cancer chemotherapy can be overcome [223,224].

#### 3.4.1. NLC-Based TKI Formulations Tested In Vitro

NLCs of different composition were prepared with the following TKIs: ERL [225], gefitinib [226], IMA [227,228], SOR [229], SUN [230] and osimertinib [231]. Their specific composition and particle size is given in Table 5. These nanoformulations demonstrated enhanced bioavailability, sustained release over several hours, and enhanced cellular uptake, which led to an increased anticancer activity resulting in overall lower dose-related side effects. An exception to the above-mentioned formulations is the work of Gorle, who did not perform in vitro test on cancer cells of his optimized final form in vitro. He designed AXT-loaded NLCs consisting of Compritol^®^ ATO 888, oleic acid and Tween^®^ 80 prepared by the high-pressure homogenization technique with average particle 202.2 nm, zeta-potential of −21.5 mV and 88% EE, exhibited in vitro a burst release of drug for the first 2 h followed by a sustained release profile for >10 h, and due to improved bioavailability of AXT reduction in dose and dosing frequency can be achieved, along with suppression of dose-related side effects [232].

#### 3.4.2. In Vivo Tested NLC-Based TKI Formulations

Nintedanib mesylate NE-loaded NLCs with mean particle size 125.7 ± 5.5 nm, zeta potential of −17.3 ± 3.5 mV and EE of 88.5 ± 2.5% releasing 6.8 ± 2.72% of drug at pH 1.2 and 92.72 ± 3.40% at pH 6.8, were efficiently taken up by Caco-2 cells and exhibited higher cytotoxicity against A549 cells compared to free drug. NE entrapped into NLCs showed even 26.31-folds higher oral bioavailability than NE suspension, but the intestinal lymphatic uptake of NLCs formulation in cycloheximide treated mice was lower than that observed with control without cycloheximide treatment [233]. IMA-loaded NLCs with mean particle size 148.80 ± 1.37 nm, zeta potential of −23.0 ± 1.5 mV, and 97.93% EE exhibited sustained drug release in vitro, pronouncedly increased drug bioavailability after i.v. and oral administration, and showed stronger in vitro cytotoxicity on human lung non-small carcinoma cells NCI-H727 compared to pure drug, particularly at doses >5 μM after 48 h incubation. Intravenous or oral administration of drug-loaded nanoformulation to rats resulted in more prolonged circulation of the drug and considerably higher mean residence time compared to free drug; pronouncedly increased bioavailability of the drug, entrapped in NLC, was reflected in 2.5-fold higher AUC_0–∞_ values compared to free drug. In addition, oral administration of IMA-loaded NLCs resulted in a 3-fold increase in C_max_ suggesting ameliorated solubility and absorption, and thus, improved bioavailability of the drug [234].

## 4. Clinical Applicability of TKI Lipid-Based Delivery Nanosystems

Lipid-based nanosystems for the delivery of several TKIs, including SNEDDSs, liposomes, SLNs, and NLCs, are discussed in detail in the manuscript. Although promising results of in vitro screening performed on various cancer cell lines have been described for all mentioned drug delivery nanosystems, the most studied are nanosystems based on liposomes and SLNs, where in vivo studies performed on mice or rats have been described. For this reason, it can be assumed that formulations based on these two nanosystems can be expected in the future in clinical trials for drug delivery enhancing stability, bioavailability, cellular absorption, and enabling sustained release and targeted delivery.

Among FDA-approved clinical trials, TKIs such as SUN, ERL, LPT, CBZ can be found, but only as drugs in approved standard formulations in combination with PTX albumin-stabilized NP formulations, see, e.g., NCT00748163, NCT00553462, NCT00733408, NCT00331630, NCT01455389, NCT00709761, NCT9031359, NCT03942068, NCT05092373 [235,236,237,238]. Therefore it can be concluded that no pure nano-TKI formulations have yet been used for clinical trials, but this fact does not exclude that they could not be successful candidates for clinical trials in the future.

## 5. Conclusions

After the discovery of imatinib, the boom of various TKIs followed, most of which are anticancer agents and some are used for reduction of inflammations (rheumatoid arthritis or lung fibrosis). However, in general, the side effects of TKIs are so serious that they are designated for only severe, progressive, debilitating, or potentially fatal conditions. TKIs are hepatotoxic; in addition, they are intensively metabolized and have limited bioavailability, and resistance to them often occurs. These negative aspects can be bridged by designing TKIs of newer generations or technological reformulation. The use of lipid-based nanoformulations have great potential for increasing drug solubility in water and oral absorption and ensuring the minimization of dose variability for patients and overall chemical stability, as well as limited metabolization (especially the so-called first pass effect). All these facts lead to a significant improvement of TKI efficacy and reduction of side effects, which makes these delivery carriers effective platforms, which are, moreover, easily prepared and highly modifiable and have high carrying capacity with the potential of passive or active targeting of cancer sites. In addition, the anticancer effect of TKIs can be improved by their co-encapsulation with an effective conventional anticancer drug. On the other hand, there are only a few in vivo studies that look into the safety of the use of these lipid-based carriers encapsulating TKIs, mechanisms of actions, and pharmacokinetic studies (absorption, distribution, metabolism, and elimination), especially in humans, to accurately determine the safety margins and parameters that should be integrated as standards for the design of new formulations that would have a chance to get into clinical studies in the near future and could provide safer and more effective systems for administration of these promising chemotherapeutics.

## Figures and Tables

**Table 1:** A brief description [27,30,31,62,63] of selected TKIs arranged alphabetically by their international nonproprietary names (INN) formulated into lipid-based nanosystems.

INN	Brand Name	Approval for the 1st Indication	Type	Kinase Target	Major Therapeutical Uses
afatinib	Gilotrif	2013	V	EGFR, HER2	NSCLC
axitinib	Inlyta	2012	II	VEGFR1-3, PDGFR	RCC
brigatinib	Alunbrig	2017	I	ALK, ROS1, IGF-1R, Flt3, EGFR	NSCLC
bortezomib	Velcade	2003	I	proteasome	multiple myeloma, MCL
cabozantinib	Cometriq, Cabometyx	2012	I	RET, HGFR, VEGFR1-3, Kit, TrkB, Flt3, Axl, Tie2, ROS1	MTC, RCC, HCC
crizotinib	Xalkori	2011	II	ALK, HGFR, ROS1, MST1R	NSCLC
dasatinib	Sprycel	2006	I	BCR-ABL, EGFR, PDGFR Src, Lck, Yes, Fyn, Kit, EphA2	CML, ALL
erlotinib	Tarceva	2004	I	EGFR, HER1	NSCLC, SCLC, PaC
gefitinib	Iressa	2009	I	EGFR	NSCLC
imatinib	Gleevec	2001	II	BCR-ABL, c-Kit, PDGFR	CML, ALL, DFSP, HES, GIST, MDS/MDP
lapatinib	Tykerb	2007	II	EGFR, HER2	breast cancer
nintedanib	Ofev	2014	I	VEGFR, FGFR, PDGFR	pulmonary fibrosis
osimertinib	Tagrisso	2015	V	EGFR	NSCLC
pazopanib	Votrient	2009	I	VEGFR1-3, PDGFR, FGFR1/3, Kit, Lck, Fms, Itk	RCC, STS
ponatinib	Iclusig	2013	II	BCR-ABL, VEGFR, PDGFR, FGFR, EphR, Src, Kit, RET, Tie2, Flt3	CML, ALL
regorafenib	Stivarga	2012	II	BCR-ABL, VEGFR, BRAF, c-Kit, PDGFR, RET, FGFR, Tie2, Eph	CRC, GIST
selumetinib	Koselugo	2020	V	MEK1/2	NF1
sorafenib	Nexavar	2005	II	B/C-Raf, BRAF, c-Kit, Flt3, RET, VEGFR1-3, PDGFR	RCC, DTC, HCC, ThC
sunitinib	Sutent	2006	II	PDGF, VEGFR1-3, c-Kit, Flt3, CSF-1R, RET	CML, RCC, GIST, PNET
trametinib	Mekinist	2013	III	MEK1/2	melanoma, NSCLC

ALL = acute lymphoid leukemia; CML = chronic myeloid leukemia; CRC = colorectal carcinoma; DFSP = dermatofibrosarcoma protuberans; DTC = differentiated thyroid carcinoma; GIST = gastrointestinal stromal tumor; HCC = hepatocellular carcinoma; HES = hypereosinophilic syndrome; MCL = mantle cell lymphoma; MDS/MDP = myelodysplastic/myeloproliferative neoplasms; MTC = medullary thyroid cancer; NF1 = neurofibromatosis type 1; NSCLC = non-small cell lung cancer; PaC = pancreatic cancer; PNET = primitive neuroectodermal tumor; RCC = renal cell carcinoma; STS = soft tissue sarcoma; ThC = thyroid cancer.

**Table 2:** In Vitro tested SNEDDS-based TKI formulations.

TKI	Ingredients	Particle Size	Tested Human Cancer Cell Lines	Benefits	Refs.
brigatinib	oleic acid, Tween^®^ 20, diethylene glycol monoethylether	ca. 50 nm	lung adenocarcinoma A549 cells	↑ solubility (205×) ↑ intestinal permeability ↑ anticancer effect	[118]
dasatinib	oleic acid, Cremophor^®^ RH-40, 1,2-propanediol	ca. 16 nm	MDA-MB-231 breast cancer cells	↑ intestinal permeability ↑ anticancer effect	[119]
imatinib	Cremophor^®^ EL, Labrasol^®^ ALF, Lauroglycol™ 90	ca. 47 nm	MDST-8 colon carcinoma cells	↑ anticancer effect	[120]
imatinib	ethyl oleate, Tween^®^ 80, polyethylene glycol 600	ca. 81 nm	MCF-7 breast cancer cells	↑ anticancer effect	[121]
sorafenib	medium-chain triglycerides, lecithin, Tween^®^ 80	ca. 43 nm	HT-29 colorectal adenocarcinoma cells	↑ anticancer effect	[122]
sorafenib	glycerol, Lipoid S75, Tween^®^ 80	75–107 nm	HepG2 liver cancer cells	↑ anticancer effect	[123]
sunitinib	Lauroglycol™ 90, Triton-X100, Transcutol^®^-P	ca. 42 nm	HT-29 colorectal adenocarcinoma cells	↑ anticancer effect	[124]

**Table 3:** In Vitro tested liposomal TKI formulations.

TKI	Ingredients	Particle Size	Tested Human Cancer Cell Lines	Benefits	Refs.
erlotinib	lecithin, cholesterol, chitosan, anti-EGFR aptamer	70–200 nm	lung adenocarcinoma PC-9, H-1975 cells	long-term stability effective targeting ↑ drug accumulation ↑ anticancer effect	[135]
imatinib	sodium-deoxycholate, hyaluronic acid, lecithin	ca. 159 nm	HT-29 colorectal adenocarcinoma cellsColo-320-DMF colon carcinoma	↑ cellular uptake ↑ anticancer effect	[136]
imatinib + paclitaxel	1,2-distearoyl-sn-glycero-3-phosphoethanolamine-*N*-[amino(polyethylene glycol)-2000], cholesterol, folic acid, phosphatidylcholine	ca. 122 nm	MCF-7 breast cancer cellsPC-3 prostate cancer cells	↑ internalization and accumulation in cancer cells ↑ anticancer effect	[137]
imatinib + tamoxifen	1,2-dipalmitoyl-sn-glycero-3-phosphocholine, monopalmitoyl-2-hydroxy- sn-glycero-3-phosphocholine, sorbitan monooleate	ca. 168 nm	MCF-7, MDA-MB-231 breast cancer cells	synergistic inhibition ↑ anticancer effect	[138]
sorafenib	maleimide-polyethylen glycol-*N*-hydroxysuccinimide, soya lecithin, trimethyl chitosan, octreotide	ca. 127 nm	HepG2 hepatocellular carcinoma cells	sustained release ↑ accumulation in cancer cells ↑ anticancer effect	[139]
sorafenib	1,2-dipalmitoyl-sn-glycero-3-phosphocholine, hydrogenated soya phosphatidylcholine, cholesterol	ca. 107 nm	renal carcinoma cells	sustained release ↑ cellular uptake ↑ anticancer effect	[140]
afatinib	1,2-distearoyl-sn-glycero-3-phosphocholine, 1,2-dioleoyl-sn-glycero- 3-phosphoethanolamine, 1,2-dioleoyl-sn-glycero-3-phosphocholine, 1,2-dioleoyl-3-trimethylammo- nium-propane chloride, cholesteryl hemisuccinate	46–57 nm	lung adenocarcinoma H-1975, H-1650, HCC-827 cells	↑ tumor-targetability induction apoptosis in H-1975 cells ↑ anticancer effect	[141]

**Table 4:** In Vitro tested SLN-based TKI formulations.

TKI	Ingredients	Particle Size	Tested Human Cancer Cell Lines	Benefits	Refs.
brigatinib	stearic acid, soya lecithin	176–787 nm	lung adenocarcinoma A549 cells	sustained release ↑ anticancer effect	[171]
gefitinib	Pluronic^®^ F127, lecithin, polyethylene glycol 2000, stearic acid, cholesterol, glucosamine	ca. 187 nm	lung adenocarcinoma A549 cells	↑ anticancer effect	[172]
erlotinib	Pluronic^®^ F68, Transcutol^®^-P, glycerol monostearate	300–800 nm	lung adenocarcinoma A549 cells	sustained release ↑ anticancer effect	[173]
erlotinib	Compritol^®^ ATO 888, Tween^®^ 80, Pluronic^®^ 407	<100 nm	lung adenocarcinoma A549 cells	↑ anticancer effect	[174]
erlotinib	1,2-distearoyl-sn-glycero-3-phosphoethanolamine-*N*-[amino(poly- ethylene glycol)-2000], hydrogenated soya phosphatidylcholine, polycaprolactone	ca. 170 nm	lung adenocarcinoma A549 cells	sustained release ↑ anticancer effect	[175]
erlotinib+ paclitaxel	Pluronic^®^ 188, methoxy- poly(ethylene glycol)-b- poly(L-aspartic acid sodium, soya lecithin, glyceryl monostearate, didodecyldimethylammonium bromide	ca. 195 nm	lung adenocarcinoma NCI-H23 cells	pH-dependent and sustained release induction of apoptosis ↑ anticancer effect	[176]
imatinib	glyceryl palmitostearate, quillaja saponin, hyaluronic acid	ca. 92 nm	MCF-7 breast cancer cells	sustained release CD44 targeting ↑ cellular uptake ↑ anticancer effect	[177]
sorafenib	1,2-distearoyl-sn-glycero-3-phosphoethanolamine-*N*-[amino(poly- ethylene glycol)-2000], folic acid, chitosan, chondroitin sulfate	ca. 178 nm	hepatocarcinoma SMMC-7721 cells	sustained release induction of apoptosis ↑ anticancer effect	[178]
sorafenib	thymidine 3′-(1,2-dipalmitoyl-sn- glycero-3-phosphate), 2′,3′-dioleyl- 5′-deoxy-5′-trimethyl-ammonium-uridine	ca. 200 nm	hepatocarcinoma HuH7, HepG2 cells breast cancer MDA-MB-134, T-47D cells	↑ anticancer effect	[179]
sorafenib	poly(D,L-lactic-co-glycolic acid), 1,2-distearoyl-sn-glycero-3-phosphoethanolamine-*N*-[amino(poly- ethylene glycol)-2000], lecithin	ca. 190 nm	breast cancer MDA-MB-231 cells prostate cancer PC3-MM2 cells	sustained release long-term stability ↑ anticancer effect	[180]
sorafenib + MK-siRNA	1,2-distearoyl-sn-glycero-3-phosphoethanolamine-*N*-[maleimide- (polyethylene glycol)-2000], cholesterol, polyethylenimine, 1-methyl-4,4-bis[(9*Z*,12*Z*)-9,12-octadecadien-1-yloxy] piperidine, egg phosphatidylcoline, SP94 targeting peptide	140–160 nm	HepG2 hepatocellular carcinoma cells	specific delivery targeting ↑ drug accumulation ↑ anticancer effect	[181]
sorafenib + selumetinib	1-palmitoyl-2-oleoyl-sn-glycero-3-phosphocholine, 1,2-distearoyl-sn- glycero-3-phosphoethanolamine- *N*-[maleimide(polyethylene glycol)-2000], poly(D,L-lactic-co- glycolic acid), polyvinyl alcohol, cholesterol, *N*-acetylgalactos- amine	ca. 170 nm	hepatocellular carcinoma HepG2, Hep3B cells glioblastoma DBTRG-05MG cells	induction of apoptosis ↑ anticancer effect	[182]
sorafenib + paclitaxel	distearoyl phosphoethanolamine-polyethylene glycol, (1,2-dipalmitoyl-sn-glycero-3-phosphoethanolamine-*N*-[methoxy- (poly(ethylene glycol)-2000], cetyl palmitate, Pluronic^®^ F68, polyvinyl alcohol	ca. 200 nm	human glioblastoma U87-MG cells lung adenocarcinoma A549 cells	↑ drug accumulation ↑ cellular uptake avoided drug efflux pumps ↑ anticancer effect	[183]
sorafenib + iron oxide	cetyl palmitate, Tween^®^ 80, EMG1300 (iron oxide with surfactant)	ca. 420 nm	HepG2 hepatocellular carcinoma cells	magnetically driven accumulation ↑ drug accumulation ↑ cellular uptake ↑ anticancer effect	[184]
sunitinib	Roghan Kermanshahi Ghee oil, fat tail sheep, dioctyl sulfosuccinate sodium salt, chitosan, gum tragacanth	110–156 nm	acute myeloid leukemia THP-1 cells	sustained release ↑ anticancer effect	[185]
sunitinib	Phospholipon^®^ 90H, soya lecithin, polyvinyl alcohol, chitosan	ca. 439 nm	MCF-7 breast cancer cells	induction of apoptosis ↑ anticancer effect	[186]
afatinib	(1,2-dipalmitoyl-sn-glycero-3-phosphoethanolamine-*N*-[methoxy- (poly(ethylene glycol)-2000], poly(D,L-lactic-co-glycolic acid), 1,2-distearoyl-sn-glycero-3-phosphoethanolamine-*N*-[methoxy- (polyethylene glycol)-2000], lecithin	147–183 nm	colorectal adenocarcinoma Caco-2 cells	↑ targeting pH-sensitive penetration ↑ cellular uptake ↑ anticancer effect	[187]
afatinib	1,2-distearoyl-sn-glycero-3-phosphoethanolamine-*N*-[carboxy(poly- ethylene glycol)-2000], 1,2-di- stearoyl-sn-glycero-3-phospho- ethanolamine-*N*-[methoxy(poly- ethylene glycol)-2000], lecithin, tight junction-modulating short peptides FD7/CCD	ca. 66 nm	lung adenocarcinoma PC9 cells	tight junctions perturbation blood-brain barrier permeation sustained release ↑ targeting ↑ anticancer effect	[188]
afatinib + cisplatin	1,2-dilauroyl-sn-glycero-3-phosphocholine, 1,2-distearoyl-sn-gly- cero-3-phosphoethanolamine- *N*-[amino(polyethylene glycol)- 2000], poly(DL-lactide- co-glycolide)	ca. 138 nm	nasopharyngeal carcinoma HONE1cells	reduced cell viability induction of apoptosis synergistic efficacy ↑ anticancer effect	[189]

**Table 5:** In Vitro tested NLC-based TKI formulations.

TKI	Ingredients	Particle Size	Tested Human Cancer Cell Lines	Benefits	Refs.
erlotinib	Precirol^®^, Miglyol^®^, poloxamer 407	ca. 109 nm	lung adenocarcinoma A549 cells	↑ cellular uptake apoptosis induction ↑ anticancer effect	[225]
gefitinib + paclitaxel	trilaurin, α-tocopherol, 1,2-distearoyl-sn-glycero-3-phosphocholine, (1,2-distearoyl-sn-glycero-3-phosphoethanolamine-*N*-[methoxy(polyethylene glycol)-2000], luteinizing hormone release hormone	100–300 nm	lung adenocarcinoma A549, H-1975, PC-9, PC-9GR cells	↑ anticancer effect	[226]
imatinib	Compritol^®^ ATO 888, oleic acid, Gelucire^®^ 48/16, Gelucire^®^ 43/01, Span 80, Tween^®^ 80	ca. 96 nm	gastric adenocarcinoma CRL-1739 cells	sustained release ↑ anticancer effect	[227]
imatinib	stearic acid, sesame oil, sodium lauryl sulphate, Tween^®^ 80	ca. 104 nm	MCF-7 breast cancer cells	↑ anticancer effect	[228]
sorafenib	tripalmitin, Captex^®^ 355 EP/NF	<300 nm	hepatocellular carcinoma HepG2, Hep3B, Huh7, PLC/PRF/5 cells	sustained release ↑ cellular uptake ↑ anticancer effect	[229]
sunitinib	Pluronic^®^ F127, cholesterol, Labrafac™, biotin, stearylamine	ca. 125 nm	lung adenocarcinoma A549 cells	sustained release (>8 h) ↑ cellular uptake ↑ anticancer effect	[230]
osimertinib	stearic acid, Labrafil^®^ M 1944CS, poloxamer 407	ca. 162 nm	lung adenocarcinoma A549 cells	sustained release ↑ permeation ↑ anticancer effect	[231]

## Data Availability

Not applicable.
